# FEM and Von Mises Analysis on Prosthetic Crowns Structural Elements: Evaluation of Different Applied Materials

**DOI:** 10.1155/2017/1029574

**Published:** 2017-04-03

**Authors:** Ennio Bramanti, Gabriele Cervino, Floriana Lauritano, Luca Fiorillo, Cesare D'Amico, Sergio Sambataro, Deborah Denaro, Fausto Famà, Gaetano Ierardo, Antonella Polimeni, Marco Cicciù

**Affiliations:** ^1^Department of Biomedical and Dental Sciences, Morphological and Functional Images, University Hospital of Messina, Via Consolare Valeria, No. 1, 98125 Messina, Italy; ^2^Department of Human Pathology, University of Messina, Messina, Italy; ^3^Department of Oral and Maxillo-Facial Sciences, Pediatric Dentistry Unit, “Sapienza” University of Rome, Rome, Italy

## Abstract

The aim of this paper is to underline the mechanical properties of dental single crown prosthodontics materials in order to differentiate the possibility of using each material for typical clinical condition and masticatory load. Objective of the investigation is to highlight the stress distribution over different common dental crowns by using computer-aided design software and a three-dimensional virtual model. By using engineering systems of analyses like FEM and Von Mises investigations it has been highlighted the strength over simulated lower first premolar crowns made by chrome cobalt alloy, golden alloy, dental resin, and zirconia. The prosthodontics crown models have been created and put on simulated chewing stresses. The three-dimensional models were subjected to axial and oblique forces and both guaranteed expected results over simulated masticatory cycle. Dental resin presented the low value of fracture while high values have been recorded for the metal alloy and zirconia. Clinicians should choose the better prosthetic solution for the teeth they want to restore and replace. Both prosthetic dental crowns offer long-term success if applied following the manufacture guide limitations and suggestions.

## 1. Introduction

Many partially edentulous patients have difficulty in functioning, speaking, chewing, and eating, leading to a decline in their quality of life. Prosthetic rehabilitation of partial edentulous jaws patients is today a common treatment that practitioners manage in their daily practice. The use of artificial crown is a method used for several years in order to replace a tooth crown affected by caries or other structural lesions. Metal-ceramic crowns are a treatment that has been, and still is, in common use for prosthetic restorations supported by natural teeth or dental implants [1, 2].

Although new ceramics such as zirconium oxide offer encouraging expectations in terms of strength and aesthetics, metal-ceramic restorations continue to be the treatment of choice in patients with parafunctional disorders and in posterior areas because of its high mechanical strength and predictability. These restorations enjoy a combination of strength and precision provided by the metal and aesthetics provided by the ceramic coating [3].

Fracture resistance is the deciding factor for determining the longevity of a restoration in the oral environment. Restorations possessing high fracture resistance have predictably high survival rates under masticatory forces [4, 5]. The rehabilitative dentistry has always paid particular attention to the detailed analysis and the application of the occlusal forces, the distribution of tensional forces, and stress dissipation, as biomechanical factors influence the prosthetic success substantially. In time, several methods have been used to study the action of the functional forces on the prosthesis and on hard and soft tissues of the oral cavity. The finite element analysis, however, is a tool that allows analytically evaluating the distribution of tensional forces at every point of the surface taken as a reference, by creating a mathematical virtual model [6, 7].

The use of finite element analysis (FEA) in dentistry rehabilitation helps understand the characteristics of the individual prosthetic dental crowns components, their physical and chemical properties, and the optimal environmental conditions because they offer the best performance.

There is a level of stress, defined as the tolerance limit, below which a biomaterial can be loaded indefinitely; that is, the structure can withstand a number of repeated load cycles over time without there being any failure by fatigue (this occurs when the rehabilitation structure is subjected to very high stresses that can be borne only for a limited number of times).

The intensity reduction of the applied loads and the number of load cycles through proper planning and the evaluation of study models of rehabilitation to be assembled will eliminate parafunctional habits and at the same time reduce the number of occlusal contacts. It means significantly to reduce the risk failure by stress [8, 9]. In this study the biomechanical behavior of prosthetics dental crowns subjected to static loads in contact with the jawbone has been highlighted.

## 2. Materials and Methods

### 2.1. The Cad Model

The tooth used in this study comes from a scan of a real M1 tooth. The scanning file was constituted by a cloud of points and provided information about the surface of the body and not about its internal composition. The recomposition of the material stratification that defines the tooth was processed in environment cad. The intermediate and superficial layers of the tooth were obtained through scaling and subtraction Boolean operations carried out in sequence; the layer of enamel thus obtained is changed from 0.9 mm to 0.3 mm (the minimum thickness is recorded on the end of the tooth), while the dentin has a thickness ranging between 1.21 mm and 0.5 mm. Also in this case the minimum thickness is recorded near the lower end of the tooth. The crown has a thickness of 0.5 mm. The innermost and the intermediate layer have been shaped for housing a common dental implant abutment.

### 2.2. The Finite Element Analysis

All the material configurations of this study were analysed by FEM analysis; the simulation platform was Ansys Workbench 17.0. 3D linear static simulations were performed.

### 2.3. Materials

In this experimental study we chose to perform a comparative analysis by comparing the results obtained considering the tooth consisting of the natural material with the results shown by the reconstructed tooth with biocompatible materials. Since the wet dentin [3, 6, 7, 9] is an anisotropic material, in this work it was decided to consider it dry; in this condition, it in fact assumes isotropic properties, cancellous bone [1, 4, 10], enamel [2, 8], Hgc-1 gold alloy [11], and zirconium carbide [12, 13].  Table 1 summarizes the mechanical properties of the materials used in each layer.

### 2.4. The Numerical Model

The realized numerical model is composed predominantly of tetrahedral elements (SOLID 187) and by a small percentage of hesaedrical elements (SOLID 186). In order to identify the best compromise between resolution of the results and computational cost, in the preliminary phase, a sensitivity mesh analysis was carried out (Tables 1, 2, and 3).

With reference to Figure 2, the red points show the mesh configuration that was chosen for the comparative analysis: the trend analysis suggests that, for further increases in the number of elements, the curve derivative is almost zero. Therefore, a thickening of the mesh would not produce significant results but increasing only the mesh density. Tables 3 and 4 summarize the details of the numerical model, including some statistics related to quality (distortion) of the elements, highlighting its suitability.

### 2.5. Loading Conditions

The simulated load condition was designed according to the testing activity performed by Wirtz et al. [14]; in particular, it was desired to reproduce the same distribution percentage of the load on the three axes, at the maximum load condition. In the image to the loading area it is highlighted.

### 2.6. Constraint Conditions and Contacts

**Figure 1:**
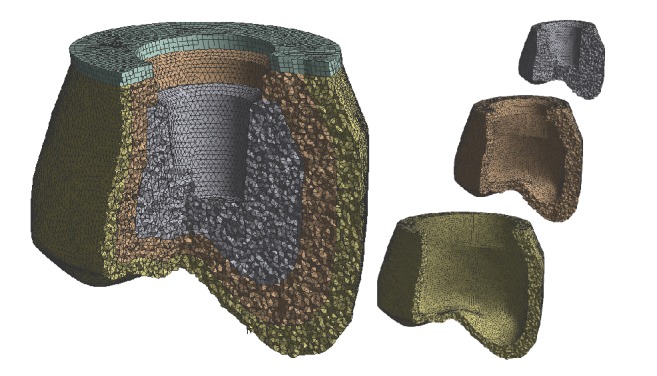
The mesh model of the dental prosthodontic crown. Shape of each material and layer.

**Figure 2:**
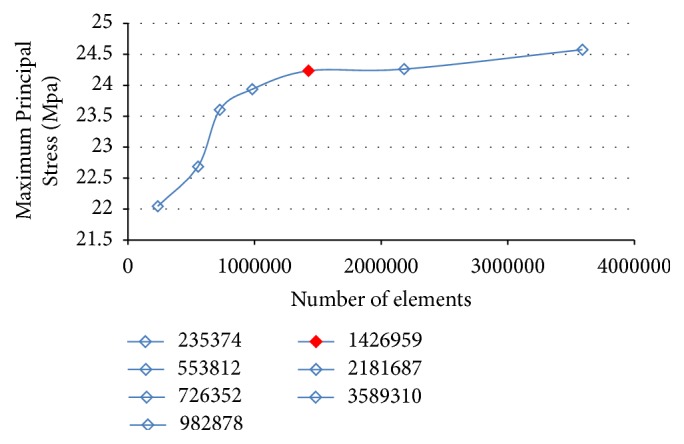
Sensitivity mesh analysis for number of elements.

**Figure 3:**
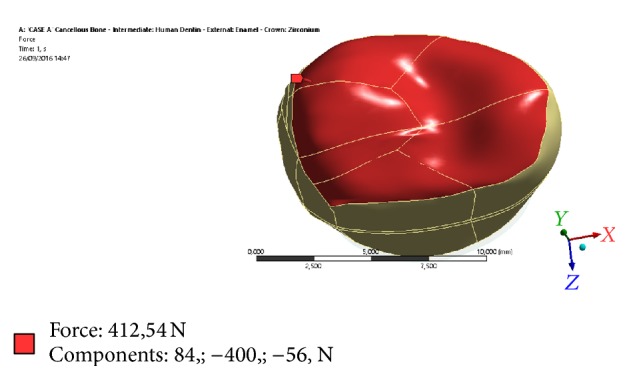
As earlier mentioned, the load chosen for the comparative analysis is not purely axial (purely axially along *y*) but shows a component along *x*-axis (21%) and a component along *y*-axis (14%).

In the FEM analysis, all the layers composing the tooth were glued together. The inner surface, wherein the implant housing is formed, was considered glued to the implant itself. The implant was considered to be rigid since its analysis is not the subject of this study. Finally, the upper surface of the crown was considered as a fixed support (Figures 1–3).

The FEM analysis carried out on the tooth modeled with natural materials was performed in the same conditions described above; although for this specific case the constraint configuration cannot be considered as realistic, it was assumed that, in order to have a reliable comparison with the models made of biomaterials, the constrain configurations should be the same.

### 2.7. Methods

The six different models were developed, which are summarized in Table 2.

## 3. Simulations and Results

The results obtained with the simulation demonstrate the relationship between the loads applied to the system, the geometrical characteristics of the materials, the constraints, and deformations. One of the theories most used to determine the stress is to apply the Von Mises law in order to underline the stress distribution over the materials and models.

**Figure 4:**
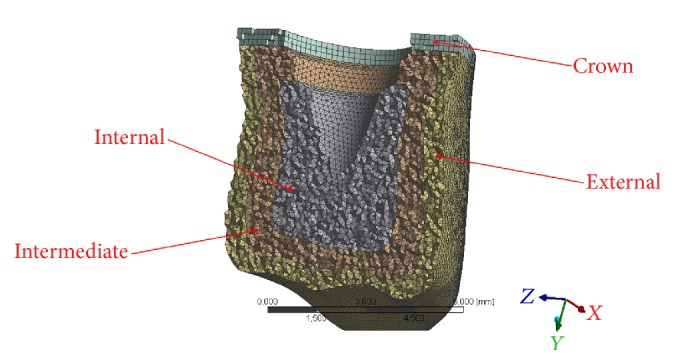
Different layers of the dental crown analyzed and divided for each material used.

**Figure 5:**
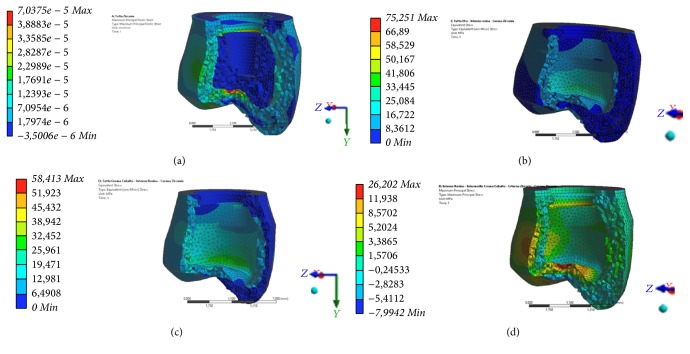
Different colors underline the stress distribution area related to the used material. The chrome cobalt alloy and the zirconium crown seem to better distribute the stress around the crown responding to vertical forces. (a) The zirconia crown distributes the stress in all the crown portions and areas even if a little stress is presented on the occlusal zone. ((b) and (c)) Chrome cobalt alloy and gold alloy guarantee perfect resistance to the fracture and a well distribution of the stress. (d) Dental resins show low resistance on fracture.

The program expresses the results in the form of a chromatic scale of colors ranging from blue to red for the minimum values for the maximum values. The values represent those of the respective solution found. The values found were compared with the critical values of the respective materials and area accordingly with the literature references information [1, 4, 10, 15, 16]. The loads, as can be noted, correspond to those obtained on the molar area for extreme operating, reported in the literature [1, 7, 16, 17]. The other types of loads are mixed and being angled with respect to the symmetry axis *Z* will generate a bending moment in the system. The influences of the soft tissues in the neighbourhood of the pillars (3 mm on thickness) are considered to be negligible, and also all the threads have been considered as horizontal and not helical (Figures 4 and 5).

Data are collected in the table for each model. For every one of the 6 models 2 photos of the elastic deformation equivalent Von Mises (whole and especially the area of maximum value) and two photographs of the voltage equivalent Von Mises (whole and especially the area of maximum value) will be reported (Table 4).

## 4. Discussion

Stress distribution around teeth and masticatory forces involving periodontal tissue and replacing dental material is a quite debated topic in the recent literature [1, 4, 7, 15–22].

The mechanical role played by crown is to start first digestion on separating food by using the enamel of the teeth cusps. Therefore, a replacement material for enamel should have a hardness value that is similar to or lower than that of enamel. A replacement material for dentin should have maximum stress, maximum strain, and elastic modulus similar to or higher than those of dentin. Several materials have been used in order to restore the dental crown restoring aesthetic and function of the damaged tooth [2, 5, 9, 23, 24].

The FEM and Von Mises parametric analyses have been recently used for creating virtual model of biomedical devices and for evaluating the stress distribution on important area. The present research is performed with model created by using engineering software according to what has been recently published in the international literature [15, 19, 21, 25].

The results of the present study clearly demonstrated what is well known in the recent literature. No one material is ideal on replacing the anatomical features of the tooth [15, 17, 19, 26].

Fabrication of provisional restorations using dental resin is an important procedure in fixed prosthodontics. Provisional resin crowns must satisfy the requirements of pulpal protection, positional stability, occlusal function, ability to be cleansed, margin accuracy, wear resistance, strength, and esthetics. They serve the critical function of providing a template for the final restorations once they have been evaluated intraorally. However, dental resins have low resistance to the fracture and according to the results of the present study they cannot be used for long masticatory cycles because of the high risk of fracture [8, 27].

The patient aesthetic request for having white metal-free and tooth-colored prosthodontics restorations has driven substantial effort to increase the strength and reliability of dental ceramic systems. According to Regish et al. [27], the therapy for restoring missing teeth has become one of the most important needs for patients attending clinics to restore esthetics or function. Many treatment modalities are available for replacing a single missing dental crown; each operative technique can be considered a predictable long-term treatment option and has its own advantages and disadvantages [9, 24, 28]. The use of gold alloy or chrome cobalt alloy has been well documented in the recent literature and the clinical results of those materials are predictable and safe. Comparing those statements with the results of the present investigations the gold alloys seem to have more resistance on the long-term stress distribution maybe for the high level of fracture, their consistency, and slow consumption. Similarly, the chrome cobalt alloy has a good resistance to the traction. Von Mises stress distribution presented similar results in both materials and no fracture for long masticatory cycle [21–23, 25, 27, 29].

From a different point of view recent studies have been directed on evaluating the consistence and the resistance of metal-free single prosthodontics crown. Even if those materials underlined excellent results for the anterior teeth, the posterior area involved in high masticatory forces resulted in fracture of the crown and in no long-term results [21–23, 29–32].

Zirconia may be considered as the most suitable substructure ceramic for anterior free contact teeth while for the posterior restorations chrome cobalt alloy seems to have chewing cycles becoming essential in predicting long-term performance before clinical usage. According to previous research published by Tsouknida et al. [33] the present model was validated and verified according to the aim of the study and subjected to a dynamic finite element analysis. Applying realistic loads and boundary conditions is a fundamental prerequisite for model reliability, as the predictive capabilities of any simulation are bound to the interacting variables considered during the analysis. Based on the foregoing setup, the developed model is accepted to provide an adequate degree of confidence for a qualitative risk estimation of the procedure variables related to the masticatory cycles. And the final purpose is not to give clinical recommendations but technical suggestion referred to a virtual model.

## 5. Conclusion 

Within the limitation of the present study, patients as well as practitioners choices regarding treatment options depend on several factors such as rejection to surgical procedures or dental implant placement, treatment duration, cost, conditions of adjacent teeth, or dental phobia. Authors presented a three-dimensional model and for this reason, additional studies are needed to guarantee lifetime of the used material and the long-term clinical success. Moreover, future therapeutic alternatives and the application of new high performance materials should be considered. Currently there is no ideal material and the investigated ones reflected different features on different load. The zirconia offers the advantages of high aesthetic but the low resistance of fracture on long-term should be considered when the restoration is about molar and posterior teeth in order to avoid the fracture during the chew.

## Figures and Tables

**Table 1:** Density of the material involved in the study.

	Enamel	Dentin	Cancellous bone	3M Filtek P60	Arcam ASTM F-75	HGC-1	Zirconium carbide
Density [kg/m^3^]	2920^*∗*^	1970^*∗*^	1200^*∗*^	?	?	17200	6645

Young's modulus [Mpa]	80000	28100	144 (*x*)	12500	241316	79000	384000
			99 (*y*)				
			344 (*z*)				

Shear modulus [Mpa]	32573	10890	53 (*XY*)	4464	92814	28214	160670
			63 (*YZ*)				
			45 (*XZ*)				

Poisson's ratio *σ*_*u*_	0,228	0,29	0,23 (*XY*)	0,4	0,3	0,4	0,195
			0,11 (*YZ*)				
			0,13 (*XZ*)				

^*∗*^Apparent density.

**Table 2:** Features of the cases presented on each material and layer.

	Materials			
	Internal	Intermediate	External	Crown
Model A	Cancellous bone	Human dentin	Enamel	Zirconium
Model B	3M-P60 resin	Zirconium	Zirconium	Zirconium
Model C	3M-P60 resin	ARCAM ASTM F-75 Chrome cobalt alloy	Zirconium	Zirconium
Model D	3M-P60 resin	3M-P60 resin	3M-P60 resin	Zirconium
Model E	3M-P60 resin	HGC-1 Dental gold alloy	HGC-1 Dental gold alloy	Zirconium
Model F	3M-P60 resin	ARCAM ASTM F-75 Chrome cobalt alloy	ARCAM ASTM F-75 Chrome cobalt alloy	Zirconium

**Table 3:** Mechanical properties from compression tests (*n* = 10 for each material) and their hardness values of dental hard tissues and dental restorative materials.

Materials	Maximum stress (MPa)	Elastic modulus (MPa)
Cortical bone	182 ± 195	70 ± 300
Medullary bone	51 ± 55	730 ± 1360
Enamel	62.2 ± 23.8	1338.2 ± 307.9^†^
Human dentin	193.7 ± 30.6	1653.7 ± 277.9^†^
Gold alloy	291.2 ± 45.3	2323.4 ± 322.4^*∗*^
Dental resin	274.6 ± 52.2	833.1 ± 92.4
Zirconia	2206.0 ± 522.9	3895.2 ± 202.9
Chrome cobalt alloy	953.4 ± 132.1	2222.7 ± 277.6^*∗*^

^*∗*^Significant difference (*p* > 0.05) based on *t*-test.

^†^Significant difference (*p* > 0.1) based on *t*-test.

**Table 4:** Mechanical properties from compression tests (*n* = 10 for each material) and their hardness values of dental hard tissues and dental restorative materials.

		A	Layer	B	Layer	C	Layer	D	Layer	E	Layer	F	Layer
Maximum Principal Stress [Mpa]	Min	−12	Ext	−6	Ext	−8	Ext	−29	Ext	−20	Ext	−10	Ext
	Max	24	Interm	31	Interm	28	Interm	39	Crown	33	Interm	32	Interm
Middle Principal Stress [Mpa]	Min	−23	Ext	−14	Ext	−17	Ext	−45	Ext	−34	Ext	−18	Ext
	Max	12	Interm	14	Interm	14	Interm	20	Interm	20	Interm	17	Interm
Minimum Principal Stress [Mpa]	Min	−94	Crown	−66	Crown	−61	Crown	−118	Crown	−85	Crown	−68	Crown
	Max	2	Interm	2	Interm	3	Interm	3	Interm	4	Interm	3	Interm
